# New Exopolysaccharides Produced by *Bacillus licheniformis* 24 Display Substrate-Dependent Content and Antioxidant Activity

**DOI:** 10.3390/microorganisms9102127

**Published:** 2021-10-10

**Authors:** Penka Petrova, Alexander Arsov, Ivan Ivanov, Lidia Tsigoriyna, Kaloyan Petrov

**Affiliations:** 1 Institute of Microbiology, Bulgarian Academy of Sciences, 1113 Sofia, Bulgaria; alexander_arsov@abv.bg (A.A.); ivanov.ivnt@gmail.com (I.I.); 2 Institute of Chemical Engineering, Bulgarian Academy of Sciences, 1113 Sofia, Bulgaria; lidinka29@gmail.com

**Keywords:** *Bacillus licheniformis*, exopolysaccharide, batch, fed-batch, antibacterial activity, antioxidant activity

## Abstract

*Bacillus licheniformis* is a soil bacterium with many industrial applications. In addition to enzymes, platform chemicals, antibiotics and phytohormones, the species produces exopolysaccharides (EPSs) of various biological activities. This study revealed that Bulgarian isolate *B. licheniformis* 24 produced EPSs consisting of galactose, glucose and mannose with substrate-dependent ratio. From glucose, *B. licheniformis* 24 secreted EPS1, consisting of 54% galactose, 39% glucose and 7% mannose. From fructose, the strain formed EPS2, containing 51% glucose, 30% mannose and 19% galactose. Batch cultivation in flasks yielded 2.2–2.6 g/L EPS1 and 1.90–2.11 g/L EPS2. Four to five times higher yields of EPS were obtained from both substrates during batch and fed-batch processes in a fermenter at 37.8 °C, pH 6.2 and aeration 3.68 vvm. The batch process with 200 g/L of starting substrates received 9.64 g/L EPS1 and 6.29 g/L EPS2, reaching maximum values at the 33rd and 24th h, respectively. Fed-batch fermentation resulted in the highest yields, 12.61 g/L EPS1 and 7.03 g/L EPS2. In all processes, EPSs were produced only in the exponential growth phase. Both EPSs exhibited antioxidant activity, but EPS2 was much more potent in this regard, reaching 811 μM Vitamin C Equivalent Antioxidant Capacity (versus 135 μM for EPS1). EPS1 displayed antibacterial activity against a non-O1 strain of *Vibrio cholerae*.

## 1. Introduction

Exopolysaccharides (EPSs) are natural nontoxic biopolymers produced by a large number of species and performing a great variety of roles [1,2,3,4]. In microbial cells, they control biofilm formation and cell growth, support the exchange of genetic information and protect microbial cells in a hostile environment. EPSs improve antigen recognition by B-lymphocytes and facilitate interaction with PRR (pattern recognition receptors) [5]. In general, bacterial polysaccharides can be capsular—tightly bound to the cell membrane or freely secreted to the culture medium. Based on their structural and chemical composition, they are divided into homo or heteropolysaccharides. To the first class belong glucans, fructans and polygalactans; to the second class—EPSs, which consist of glucose, galactose, rhamnose, *N*-acetyl glucosamine, *N*-acetyl galactosamine, glucuronides and various derivatives of these subunits containing phosphates, glycerol or acetyl groups [5,6,7,8]. Hydrophilic EPSs have a high ability to retain water, thus maintaining a hydrated microenvironment and allowing survival. They may also form a hydrated polymer network, mediate the mechanical stability of biofilms and serve as a source of carbon, nitrogen and phosphorus-containing compounds for use by the biofilm ecosystem. EPSs can promote hydrophobic interactions and adherence to solid surfaces [9].

In recent years, bacterial EPSs have found extensive applications as ingredients of pharmaceuticals, nutraceuticals and functional foods, cosmetics and insecticides. Up to now, polysaccharides from plants and algae have dominated the market, but there are indications that microbial EPSs may change that soon [3,10]. The industrial application of bacterial EPSs is less due to their physical and chemical properties than to their unique biological activities. The intense scientific attention paid to microbial EPSs is stimulated by their potential health-promoting effects [5,11,12] since they possess immunostimulatory [13,14], antiviral, antibacterial and anticancer activity [14,15,16,17].

A recently discovered property of EPSs of particularly important applications in medicine and the food industry is their action as scavengers of reactive oxygen species (ROS). ROS are a diverse group of unstable and highly reactive oxygen-derived molecules, such as hydrogen peroxide (H_2_O_2_), hydroxyl radical (•OH), singlet oxygen (^1^O_2_) and superoxide (O_2_^−^). Their high production under stress conditions leads to destructive oxidation of macromolecules (lipids, proteins, DNA) and the disruption of various redox signalling pathways in the eukaryotic cells. Cardio-vascular diseases have been most widely studied in this context, but oxidative stress (caused by the abnormally high levels of ROS) has also been implicated in diabetes, neurologic and inflammatory diseases, various types of cancer, ageing [18,19,20] and autoimmunological disorders [21]. EPSs produced by plant-promoting rhizobacteria have been reported to increase the production of antioxidant enzymes such as superoxide dismutase (SOD), peroxidase and catalase [22]. EPSs with remarkable antioxidant properties, effectively acting as scavengers of various ROS, are produced by the bacterial species *Deinococcus radiodurans* and *Paenibacillus mucilaginosus* [2,6].

*B. licheniformis* is a Gram-positive, motile, non-pathogenic bacterium ubiquitously spread in natural habitats. As it is associated with plant material, it can be easily isolated from soil, water and the rhizosphere [1,23,24,25,26], but it can also be found on the feathers of terrestrial and aquatic bird species, and in marine water [27]. *B. licheniformis* is widely used in industry, horticulture and pharmacy. It is a promising producer of enzymes, platform chemicals [28,29,30], antibiotics [31,32] and EPSs with antimicrobial, antioxidant and anticancer activities [33,34]. That is why the species is commonly used in the biocontrol of plant diseases [35] and the development of probiotics [36,37].

The strain *B. licheniformis* 24 used in this study was previously isolated in Bulgaria and was tested for the production of 2,3-butanediol from renewable substrates [28,38,39]. During these studies, its high potential to produce EPSs was observed. The present work aims to study the EPSs formed by *B. licheniformis* 24 with a focus on their content and biological activity. The influence of the substrate-dependent content of the EPSs and the effect of the batch and fed-batch fermentation regime on the final yield are discussed below.

## 2. Materials and Methods

### 2.1. Bacterial Strains, Media and Chemicals

*B. licheniformis* strain 24 was isolated from a soil sample, which was taken on 06.03.2018 near the bed of Yantra River in Veliko Tarnovo Province, Bulgaria (43°04′52.46″ N 25°37′44.54″ E) at air temperature 10 °C. The purified strain 24 was identified by 16S rDNA sequencing. The sequence was deposited in NCBI GenBank database under accession no. MK461938 [28]. The strain is stored in the microbial culture collection of the Institute of Microbiology, Bulgarian Academy of Sciences, frozen at −80 °C, in a nutrient medium supplemented with 20% glycerol.

Two of the reference strains used in the antibacterial activity assay, *Escherichia coli* NBIMCC 3397 and *Pseudomonas aeruginosa* NBIMCC 1390, were purchased from the National Bank for Industrial Microorganisms and Cell Cultures, Bulgaria. *Klebsiella pneumoniae* G31 (NBIMCC 8650) and *Vibrio cholerae* non-O1 strain V13 (NBIMCC 8715) were isolated and identified previously and deposited in the same culture collection [40,41].

The fermentation medium (FM) for EPS production was initially developed for *Paenibacillus polymyxa* by Okonkwo et al. [42] and optimized for *B. licheniformis* 24 by Tsigoriyna et al. [39]. FM had the following content (g/L): glucose (fructose), either 50 or 100; yeast extract, 13.38; tryptone, 6.41; (NH_4_)_2_SO_4_, 1; KH_2_PO_4_, 3.5; K_2_HPO_4_, 4.2; MgSO_4_, 0.32; ammonium acetate, 2.5; CoCl_2_ × 6H_2_O, 0.09; microelements solution, 3 mL per litre, containing (g/L): FeSO_4_, 0.4, H_3_BO_3_, 0.8; CuSO_4_ × 5H_2_O, 0.04; NaMoO_4_ × 2H_2_O, 0.04; MnCl_2_ × 4H_2_O, 5.0; ZnSO_4_ × 7H_2_O, 0.1; Co(NO_3_)_2_ × 6H_2_O, 0.08; CaCl_2_ × 2H_2_O, 1.0; Biotin, 0.01.

*E. coli*, *K. pneumoniae* and *P. aeruginosa*, used in the assays for the antibacterial activity, were grown in Luria–Bertani (LB) medium (10 g/L tryptone, 5 g/L yeast extract, 10 g/L NaCl). *Vibrio cholerae* was cultured in Nutrient broth (g/L): peptone, 15; yeast extract, 3; NaCl, 6; D(+)-glucose, 1. The media were supplemented with 15 g/L agar when needed.

Glucose, xylose, galactose, raffinose, rhamnose, arabinose, fucose, mannose and fructose, which were used for HPLC standards, and the other chemicals, were of analytical grade and purchased from Merck KGaA, Darmstadt, Germany.

### 2.2. Batch Cultivation in Flasks without pH Control

The experiments for EPS production were performed in 500 mL Erlenmeyer flasks (Boeco, Hamburg, Germany), with 100 mL media containing 50 or 100 g/L glucose or fructose. Overnight culture grown in FM containing 20 g/L glucose or fructose was used as inoculum (10%). Fermentations were performed at 37 °C, and 140 rpm in GFL 1092 rotary shaker (GFL Gesellschaft für Labortechnik mbH, Burwedel, Germany). Samples from the supernatants were analysed after centrifugation at 12,000× *g* for 10 min on Model 1–14 centrifuge (Sigma, Osterode am Harz, Germany). Three independent cultivations were carried out.

### 2.3. Batch and Fed-Batch Processes with pH Control

Batch and the fed-batch fermentations were performed in Biostat^®^ Aplus fermenter with working volume 1 L (Sartorius Stedim Biotech, Gottingen, Germany) at parameters previously optimized for *B. licheniformis* strain 24: temperature 37.8 °C, pH 6.23 and aeration flow 3.68 vvm [39]. Batch processes were carried out using FM supplemented with 200 g/L glucose and 10% inoculum (grown for ~24 h). An additional air pump and rotameter were used to increase the airflow supply. The pH was controlled by the addition of 6M NaOH or 5M HCl. In fed-batch fermentation, sterile sugar solutions (700 g/L glucose and 870 g/L fructose) were added. The addition times were at 40th h (glucose), and at 28th h, 36th h and 54th h (fructose). The experiments were duplicated.

### 2.4. EPS Purification and Hydrolysis

EPSs formed from glucose (EPS1) and fructose (EPS2) were extracted by the following procedure. Crude EPS fractions were isolated from the fermentation medium after initial centrifugation at 6000× *g* for 30 min to remove the biomass. Then, the supernatant was deproteinated by incubation with 14% trichloroacetic acid (Merck KGaA, Darmstadt, Germany) in a rotary shaker (90 rpm), at 37 °C for 40 min. Then, the sample was centrifuged at 10,000× *g* for 10 min, at 4 °C, to remove the denatured proteins. The supernatant (crude EPS) was precipitated against three volumes of ice-cold ethanol (96%) and incubated at −18 °C overnight. The EPSs were harvested by centrifugation at 10,000 × *g* for 20 min, washed twice with 50% ethanol, the pellet was air-dried and dissolved in sterile bidistilled water. After overnight dialysis at 4 °C, the sample was dried in a desiccator and stored at 4 °C. Bradford assay showed that both EPSs did not contain any amounts of residual protein.

The EPS hydrolysis was performed as 10 mL solution with a concentration of 1 mg/mL was carefully mixed with 1.1 mL concentrated HCl and incubated in a sealed ampoule at 100 °C for 3.5 h. Then the hydrolysate was lyophilised by the use of a vacuum freeze dryer machine, model BK-FD10S (Biobase, Jinan City, Shandong, China). The cold trap temperature was −56 °C, the condenser temperature −80 °C and the freeze-drying time was 24 h. The lyophilised sample was dissolved in 1 mL sterile bidistilled water.

### 2.5. Antibacterial Activity

The antibacterial activity of EPS was measured by the agar well diffusion method as previously described [43].

Briefly, 100 μL of sterile water-diluted EPS with concentrations 1, 5 and 10 mg/mL were placed in previously prepared wells (10 mm in diameter) solidified by addition of 1.5% agar medium, appropriate for the respective test microorganism and containing the test culture grown for 24 h, diluted to 10^6^ CFU/mL. Then, agar plates were cultivated for 24 h at 37 °C. The antibacterial activity was estimated according to the measurement of the growth inhibition zone around the agar well in millimetres. As positive control, chloramphenicol solution with concentration 25 μg/mL was used; as negative control—the respective nutrient broth. The experiments were repeated twice.

### 2.6. Antioxidant Activity

The antioxidant activity of EPSs was estimated by Bioquochem S. L. (Llanera, Asturias, Spain) by several different methods [44,45]. The reduction of the organic cation radical 2,20-azino-bis (3-ethylbenzothiazoline-6-sulfonic acid) (ABTS) and the decrease in its absorbance at 734 nm was used to evaluate the Vitamin C Equivalent Antioxidant Capacity (VCEAC, μM). EPSs with concentrations of 100 mg/L were used.

Total Antioxidant Capacity (TAC) was determined by an electrochemical method based on oxidising the sample and detection of the released electrons with a special portable device. For eBQC values estimation, eBQC Natural Ingredients apparatus (eBQC NI) was used. EPSs were of concentrations 500 mg/L.

SOD Activity Assay Kit was used to ascertain the ability of the EPS to scavenge superoxide radicals. Briefly, the method is colorimetric and based on the reduction of a tetrazolium salt (WST-1) to formazan by the superoxide radical. SOD inhibits the reduction of WST-1 by scavenging superoxide. The inhibition was measured by the decreased absorbance at 430 nm after incubation for 20 min at 37 °C. One unit of SOD is defined as the amount of enzyme necessary for 50% dismutation of the superoxide radical. EPSs with concentrations of 100 mg/L were used for this method. All experiments for the antioxidant activity estimation were triplicated.

Hydroxyl Radical Antioxidant Capacity (HORAC) was measured by the oxidation of fluorescein and the quenching of its fluorescence (excitation 485 nm, emission 528 nm). EPSs were diluted until concentration 500 mg/L.

### 2.7. Analytical Methods

The cell growth was estimated by viable cells counts (CFU, colony-forming units, per mL) of decimal dilutions of samples, which were grown on Luria–Bertani (LB) agar plates.

The carbohydrate content in EPS was tested with the phenol-sulfuric acid colorimetric method of Dubois [46], following the procedure as described by Nielsen [47]. The protein concentrations were estimated by the Bradford method [48].

The sugar content of acid-hydrolysed EPSs was analysed by YL Instrument 9300 HPLC System (YL Instrument Co., Ltd., Anyang, Korea). Monosaccharides were separated in two different ways by the use of two different HPLC columns: Aminex HPX-87P and HPX-87C (Bio-Rad Laboratories, CA, USA). HPX-87P column was used for the xylose, galactose and mannose, while HPX-87Cwas used for mannose and fructose separation. In both analyses, the column temperature was set at 85 °C, and water with a flow rate of 0.6 mL/min was used as a mobile phase. All compounds were detected by the RI detector (YL 9170 RI Detector).

## 3. Results

### 3.1. Selection of EPS Producing Strain B. licheniformis 24

**Figure 1:**
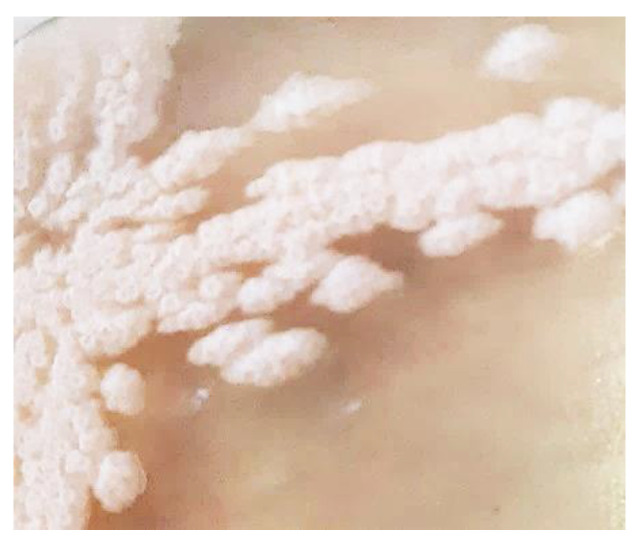
Colonies of *B. licheniformis* 24 on Luria–Bertani (LB) agar medium.

*B. licheniformis* 24 was selected from a large *Bacillus* collection based on the phenotypic characteristics of the colonies (Figure 1), and the typical slimy growth in the liquid medium.

**Figure 2:**
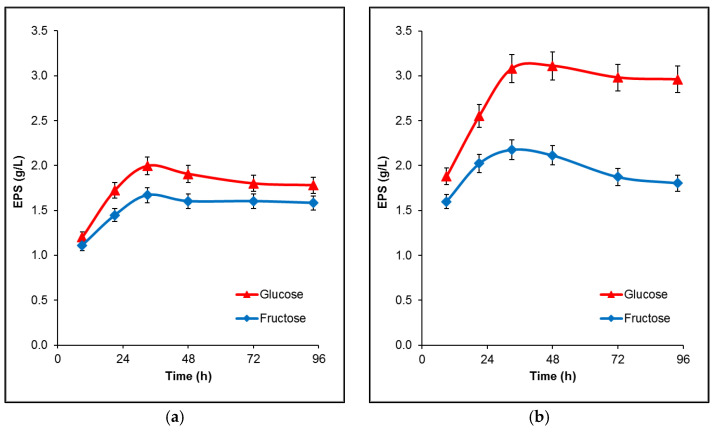
Time profiles of EPS production by *B. licheniformis* 24 during fermentation in flasks. (**a**) EPS production from glucose or fructose with initial concentration 50 g/L; (**b**) EPS production from glucose or fructose with initial concentration 100 g/L. The mean values from three independent cultivations are presented.

The initial study of the strain’s ability to produce extracellular polysaccharides was performed in flasks, in a fermentation medium containing 50 and 100 g/L glucose or fructose as a carbon source. EPS production kinetics are shown in Figure 2. *B. licheniformis* 24 was able to synthesize more exopolysaccharides from glucose than from fructose at both sugar concentrations, reaching a maximum at 33 h of cultivation. The higher concentration of the carbon sources significantly increased the difference in the conversion between them, leading to higher amounts of both EPS: 3.11 and 2.17 g/L from glucose and fructose, respectively (Figure 2b).

### 3.2. Isolation of Exopolysaccharides and Determination of Their Monosaccharide Content

**Figure 3:**
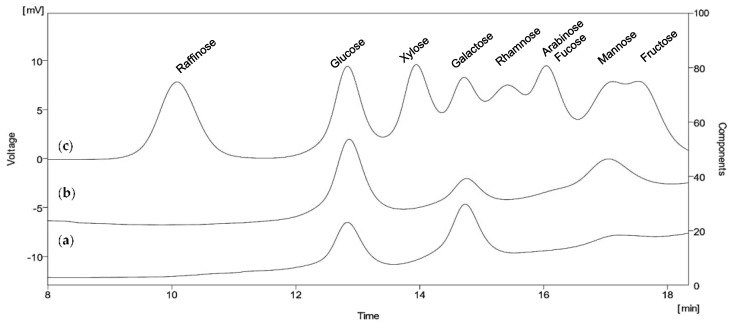
HPLC profiles of monosaccharide content of acid-hydrolysed EPSs produced by *B. licheniformis* 24. The sugars were separated by Aminex HPX-87P column and detected by RI detector. (**a**) Sugar profile of EPS1 produced from glucose; (**b**) Sugar profile of EPS2 produced from fructose; (**c**) Referent sugar standards.

The results presented in Figure 3 reveal that both EPSs consisted of the following three monosaccharides: glucose, galactose and mannose. The ratios, however, were completely different. EPS1, synthesized from glucose as a carbon source, consisted mainly of galactose (54%) and glucose (39%), while mannose was found in it in a minimal amount (7%). More than half of the total sugar content of EPS2, synthesized in a fructose-containing medium, was glucose (51%), followed by mannose (30%) and galactose (19%).

Interestingly, the composition of both exopolysaccharides lacked fructose, despite the intensive growth and consumption of fructose by the strain. Other sugars typically reported in the EPS of *B. licheniformis*, such as ribose, rhamnose, arabinose, fucose and the amino-sugars N-acetyl galactosamine or N-acetylglucosamine, were not detected too.

### 3.3. Enhanced EPS Synthesis in Batch and Fed-Batch Processes

**Figure 4:**
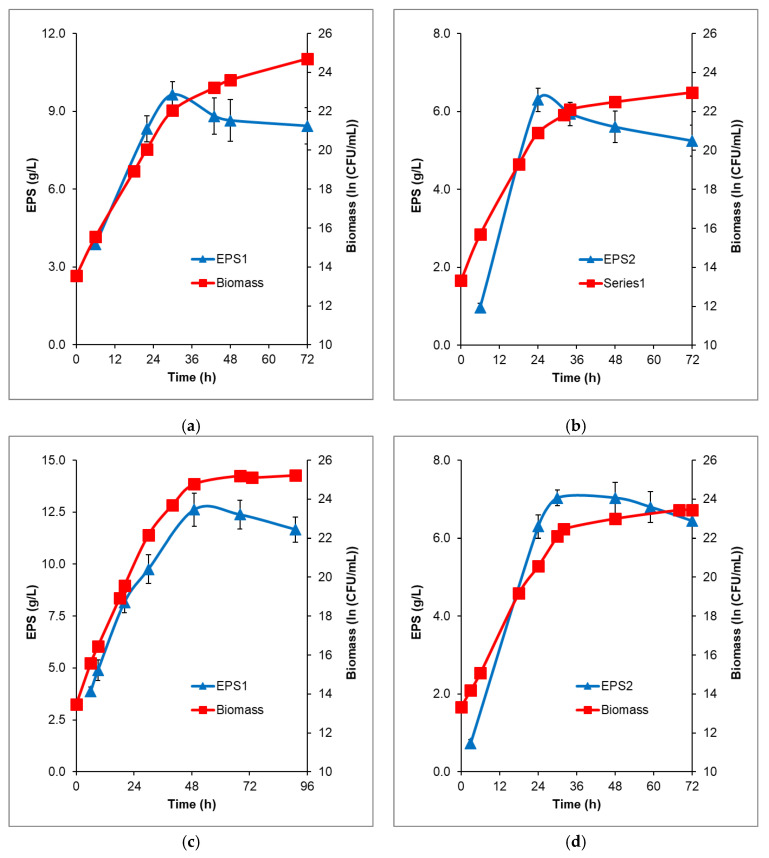
Time profiles of biomass formation and EPS production by *B. licheniformis* 24 during batch and fed-batch fermentation in fermenter Biostat^®^ Aplus, working volume 1 L. (**a**) EPS1 production during batch fermentation of glucose; (**b**) EPS2 production during batch fermentation of fructose; (**c**) EPS1 production during fed-batch fermentation of glucose; (**d**) EPS2 production during fed-batch fermentation of fructose. The starting sugar concentrations were 200 g/L; sterile glucose or fructose was added during the fed-processes—125 mL glucose (700 g/L) and 210 mL fructose (870 g/L). The mean values from two independent batch and fed-batch processes are presented.

To increase the EPSs concentrations obtained from each carbon source, batch fermentations with 200 g/L initial glucose or fructose were performed in a stirring fermenter with an additional air supply. The time profiles of EPS production from glucose and fructose followed similar kinetics, reaching a maximum and then constantly decreasing, which indicates that *B. licheniformis* 24 consumes some part of the EPS (Figure 4).

Fructose was converted more rapidly, with EPS2 reaching the maximal amount earlier (6.29 g/L at the 24th h) compared to EPS1 from glucose (9.64 g/L at the 30th h). However, the quantity of EPS1 was 1.5-fold higher and remained more stable in time.

To estimate the maximum EPS production capacity of *B. licheniformis* 24 at constant carbon source excess, starting with 200 g/L glucose or fructose, fed-batch processes were carried out. The addition of the respective carbon source in the course of the fermentation provided a consistently high substrate concentration. Thus, the highest concentrations of the produced polysaccharides were achieved: 12.61 g/L from glucose (49th h) and 7.03 g/L from fructose (30th h), (Figure 4d).

A comparison of EPSs production during batch and fed-batch processes is shown in Table 1. The process productivities and yields are higher in batch fermentation; however, the highest amounts of the produced EPS were obtained during fed-batch processes.

### 3.4. Antioxidant Activity of EPS Produced by B. licheniformis 24

**Figure 5:**
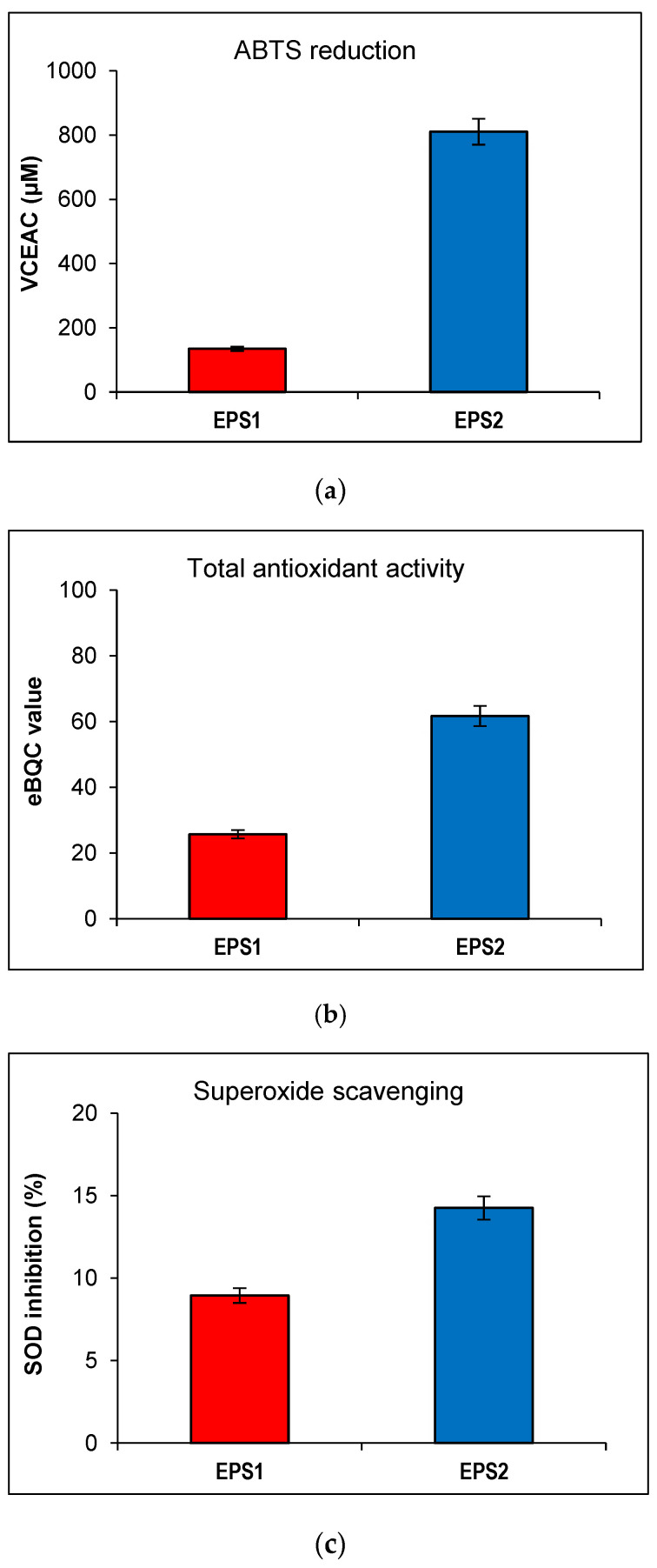
Antioxidant activity of EPS1 and EPS2 from *B. licheniformis* 24. (**a**) ABTS reduction measured in VCEAC (μM); (**b**) total antioxidant activity in eBQC values; (**c**) superoxide scavenging measured in % SOD activity inhibition. All measurements were performed in triplicates.

The strongest difference between the two EPSs was observed in their capacity to reduce ABTS, a free radical and a green chromophore with an absorbance peak at 734 nm commonly used to estimate general antioxidant activity. EPS2 showed 58.58% decreased absorbance and 810.56 μM VCEAC. In contrast, EPS1 reached only 7.27% decreased absorbance and 134.55 μM VCEAC, eight and six times, respectively, lower than EPS2 (Figure 5a).

This result was confirmed with the eBQC Natural Ingredients portable device. EPS2 showed 2.4 times higher total antioxidant activity compared to EPS1 (61.67 vs. 25.67 eBQC values) (Figure 5b). A less pronounced effect in the same direction was observed regarding superoxide scavenging. The inhibition of SOD activity proved to be weak in both EPSs, but was 5% better in EPS2 (14.25%) compared to EPS1 (8.94%) (Figure 5c). Regarding this result, the HORAC test for hydroxyl radicals was also performed, but the absence of a dose-dependent response (data not shown) suggested that the pH of the samples have an undesirable influence.

### 3.5. Antimicrobial Activity of EPS Produced by B. licheniformis 24

**Figure 6:**
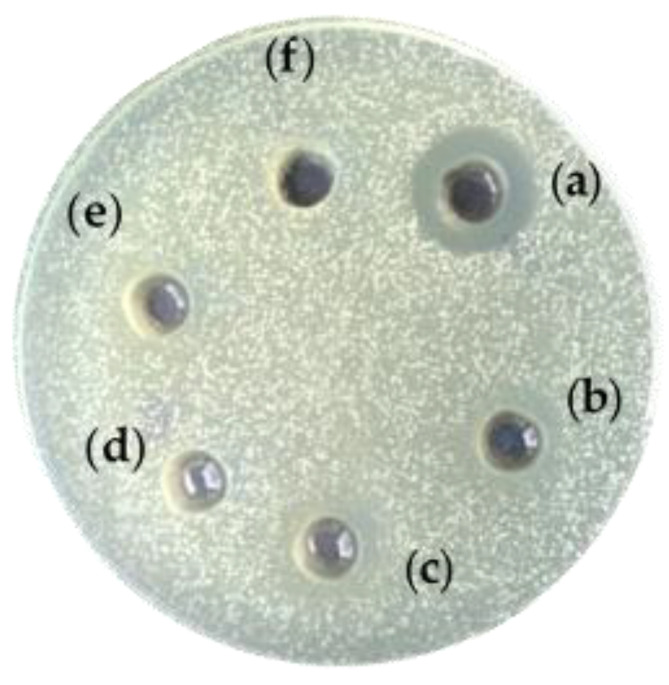
Antimicrobial activity of EPS from *B. licheniformis* 24 against *Vibrio cholerae* non-O1 strain. (**a**) EPS1, 10 mg/mL; (**b**) EPS1, 5 mg/mL; (**c**) EPS1, 1 mg/mL; (**d**) EPS2, 10 mg/mL; (**e**) EPS2, 5 mg/mL; (**f**) EPS2, 1 mg/mL.

The antimicrobial activity of the EPS against pathogenic bacteria of the species *E. coli* NBIMCC 3397, *Pseudomonas aeruginosa* NBIMCC 1390, *Klebsiella pneumoniae* G31 and *Vibrio cholerae* non-O1 strain V13 was checked. Unexpectedly, EPS1 and EPS2 in concentrations between 1 and 10 mg/mL showed no activity against most of the pathogens. However, EPS1 showed a marked, dose-dependent response against *Vibrio cholerae* non-O1 strain at concentrations of 1, 5 and 10 mg/mL (Figure 6), forming a 12 to 20 mm growth inhibition zone. The minimal inhibitory concentration was 1 mg/mL, with no bactericidal effect. EPS2 showed no activity under the same conditions.

## 4. Discussion

The present work reveals the ability of *B licheniformis* strain 24 to synthesize large amounts of two different exopolysaccharides. The EPSs we have obtained are heteropolysaccharides with a substrate-dependent specific content of galactose, glucose and mannose. In the literature, *B. licheniformis* is distinguished by the rich spectrum of sugars in EPSs composition sometimes reaching up to eight different monosaccharides, glucuronic and galacturonic acids [33,34]. However, EPSs containing only a galactose/mannose/glucose combination has been found in only one other *Bacillus* sp. [49], although it occurs regularly in EPSs produced by lactic acid bacteria [50,51,52]. Mannose content of 30% in EPS2 is the highest reported so far and is significantly greater than 1.4% reported for other similar EPSs of *B. licheniformis* [49]. EPSs high in mannose are particularly desired in the production of functional foods as strong antioxidant agents [5]. Recently, Zhu et al. [53] reported that EPSs rich in mannose produced by *Weissella cibaria* exhibited high antioxidant activities in vitro.

However, unlike lactic acid bacteria, which produce such EPSs in very limited quantities, *B. licheniformis* synthesizes and secretes them in high concentrations in the culture medium. Because of its Generally Regarded as Safe (GRAS) status, *B. licheniformis* may be considered an important industrial source of EPS as these can be freely and safely added to drugs, foods, cosmetics and other goods to improve their rheological and functional properties. In addition, given that the price of a product obtained by biotechnology is largely determined by the fermentation substrates, *B. licheniformis* is likely to be competitive in the industrial market. As this species utilises a huge range of carbohydrates, EPSs could be produced from cheap, renewable and affordable substrates such as starch, inulin and molasses, and even lignocellulosic hydrolysates [54].

The production of EPS is influenced by several factors, some of which have been studied in our work. For example, some authors believe that the nature of the substrate is irrelevant to the EPSs obtained. We show here that this is of great importance for *B. licheniformis* as two different EPSs, with completely different ratios between the composing sugars, are formed from glucose and fructose. Both EPSs were produced during the exponential growth phase and slightly decreased in the stationary phase, regardless of continuing biomass accumulation. The only requirement for this EPS production is that the substrate should be in excess, as mentioned by Freitas et al. [3]. Therefore, fed-batch experiments were performed to achieve maximal EPS production (12.61 g/L for EPS1 from glucose and 7.03 g/L for EPS2 from fructose). Both concentrations are among the highest yet reported for *B. licheniformis* (Table 2).

Other important factors for the production of EPSs from bacilli are aeration and pH. Freitas et al. [3] mentioned that microaerophilic conditions may be beneficial to EPS synthesis. In the case of *B. licheniformis*, fermentation in fully aerobic conditions favours the production of EPSs. The pH was selected as the most suitable for *B. licheniformis* growth. A pH of above 6, along with high aeration (3.68 vvm), was found to ensure the most efficient EPS synthesis.

EPSs produced by the probiotic *Lactobacillus* and *Bacillus* strains showed remarkable antimicrobial properties [60]. Such examples are the EPS of *L. rhamnosus* (isolated from breast milk), active against pathogenic *E. coli* and *Salmonella enterica* serovar *Typhimurium* [61], the EPS of *Lactobacillus gasseri* that inhibited *Listeria monocytogenes* [51], and *B. licheniformis* strain Dahb1 (isolated from shrimp intestine), active against *Pseudomonas aeruginosa*, *Proteus vulgaris*, *B. subtilis*, *B. pumilus* and *Candida albicans* [62]. To our knowledge, this is the first report on EPS inhibition of the growth of *Vibrio cholerae*, a pathogen that is very common in nature and causes 4 million cases, and up to 143,000 deaths due to cholera worldwide [63]. The high galactose content (54%) may contribute to the antimicrobial activity of EPS1 against *V. cholerae*, although the only galactose-rich EPS with an antimicrobial activity until now is the antifungal EPS of *Lacticaseibacillus rhamnosus* inhibiting *C. albicans* and *C. glabrata* [60].

Bacterial EPS with antioxidant activity have been reported in many species present in probiotics and functional foods, for example, *Enterococcus faecium* WEFA23, *L. gasseri* FR4, *Lactiplantibacillus plantarum* JLAU103, *Weissella cibaria* SJ14 and *Bifidobacterium bifidum* WBIN03 [5,50,51,52,53,64]. Regarding *B. licheniformis*, antioxidative EPSs are also usually produced by food-derived or probiotic strains, such as probiotic *B. licheniformis* AG-06 from Indian polyherbal traditional medicine [33], the strain S-1 from Sichuan pickles [49], and KS-17 and KS-20 isolated from Kimchi [65]. Both EPSs in our study showed antioxidant activity, with EPS2 achieving six times more powerful reduction of ABTS. VCEAC value of EPS2 (810 μM) corresponds to 71% of the antioxidant capacity of Vitamin C. This result is similar to the antioxidant capacity effect of EPS isolated from probiotic *B. licheniformis* by Vinothkanna et al. [33], even at a ten-fold lower concentration (0.1 mg/mL). However, regarding superoxide radicals scavenging by EPS1 and EPS2, the difference was only about 5%, which indicates that their antioxidative activity probably targets different types of ROS.

Considering the kinetics and concentrations reported above from fed-batch fermentation, EPS2 can be easily produced and consumed in amounts to match the Vitamin C content of citrus fruits such as orange (71 mg/100 g fresh mass) and mangaba (*Hancornia speciosa*, 96 mg/100 g fresh mass). It should be noted, however, that the correlation between Vitamin C content and overall antioxidant activity is far from straightforward. For instance, the fruit of murici (*Byrsonima crassifolia*) contains only 11.8 mg of Vitamin C per 100 g fresh mass, and yet showed the highest antioxidant activity (more than 30% higher than that of the mangaba) according to a study of exotic fruits from north-eastern Brazil [66].

## 5. Conclusions

The present study reports the ability of *Bacillus licheniformis* strain 24 to produce high amounts of EPSs in a substrate-dependent manner. Two different EPSs were obtained when glucose or fructose was used as a carbon source. Fed-batch fermentation led to the highest production: 12.61 g/L for EPS1 from glucose and 7.03 g/L for EPS2 from fructose; both yields were among the highest yet reported for *B. licheniformis*. EPS1 and EPS2 were found to consist primarily of galactose (51%) and glucose (51%), respectively, with the major difference in the mannose content as well (30% for EPS2 vs only 7% for EPS1). This composition probably determines the biological activity of EPSs, which displayed antimicrobial (EPS1) and antioxidant properties. EPS1 showed growth inhibitory activity against *Vibrio cholerae* non-O1 strain. EPS2 showed a significant antioxidant activity corresponding to 71% of the antioxidant capacity of Vitamin C. In conclusion, as an EPS producer, *B. licheniformis* strain 24 possessed the following advantages: it produces EPSs in high quantity, with high antioxidant activity, and is a non-pathogenic microorganism. Thus, the present study opens up possibilities for EPSs derived from *B. licheniformis* to be used as the ingredients of functional foods and other health-promoting substances.

## Figures and Tables

**Table 1:** Comparison of EPS produced by *B. licheniformis* strains in batch and fed-batch processes from glucose and fructose. The average values of two distinct experiments are presented.

Substrate/Mode	EPS (g/L)	EPS (g/L/h)	Y_EPS_^a^ (mg/g)	Biomass ^b^ (CFU/mL)	Substrate Consumption Rate (g/L/h)
Glucose					
Batch	9.64 ± 0.53	0.321	75.3	1.9 × 10^9^	4.09
Fed-batch	12.61 ± 0.79	0.257	62.9	4.3 × 10^9^	4.30
Fructose					
Batch	6.29 ± 0.33	0.262	44.1	1.1 × 10^9^	
Fed-batch	7.03 ± 0.18	0.234	37.6	4.1 × 10^9^	5.94

^a^ Milligram produced EPS per gram substrate consumed; ^b^ Biomass at the EPS maximum.

**Table 2:** Comparison of EPS produced by *B. licheniformis* strains.

Strain	Carbon Source	EPS Yield	Main Sugars in EPS Composition and Their Ratio	Reference
*B. licheniformis* AG-06	Sucrose (20 g/L)	0.56 g/L	galactose/rhamnose/xylose/mannose/glucose; 32/29/7/15/17	[33]
*B. licheniformis* T8	Monosodium Glutamate (10.0 g/L)	3.07 g/mL	mannose/ribose/glucuronic acid/galacturonic acid/glucose/galactose/arabinose/fucose; In BL-P1: 4.07/0.34/0.05/0.04/0.00/4.27/0.47/0.04/0.04/0.05; In BL-P2: 11.95/0.53/0.07/0.23/0.01/0.89/3.97/0.04/0.07/0.20	[34]
*B. licheniformis* M4	Molasses (20 mL/L)	9.0 g/L	n/a	[54]
*B. licheniformis* B22	Glucose (2.5 g/L); Alginic acid (1 g/L)	0.67 g/L	glucose/arabinose/xylose	[55]
*B. licheniformis* SVD1	Sucrose (40 g/L)	1.9 g/L	CEPS ^a^: galactose; EPS1: fructose; EPS2: mannose/galactose; unknown ratio	[56]
*B. licheniformis* MS3	Mango peels (SSF ^b^)	15.6 g/L	mannose/glucose/fructose; 20.6/46.8/32.58	[57]
*B. licheniformis* DM-1	Sucrose (20 g/L)	1.29 g/L	glucose/mannose/galactose ^c^	[58]
*B. licheniformis* T14	Sucrose (50 g/L)	0.37 g/L	fructose/fucose/glucose; 1.0/0.75/0.28	[59]
*B. licheniformis* 24	Glucose	12.61 g/L	galactose/glucose/mannose; 54/39/7	This study
*B. licheniformis* 24	Fructose	7.03 g/L	glucose/mannose/galactose; 51/30/19	This study

^a^ Capsular polysaccharide; ^b^ SSF—Solid State Fermentation; ^c^ Proteoglycan containing 67.4% (*w*/*w*) sugar and 27.6% (*w*/*w*) protein.

## Data Availability

Not applicable.

## Sample Availability

*B. licheniformis* 24 is available from the authors.

## References

[1] Mahdi I., Fahsi N., Hafidi M., Allaoui A., Biskri L. (2020). Plant Growth Enhancement using Rhizospheric Halotolerant Phosphate Solubilizing Bacterium *Bacillus licheniformis* QA1 and *Enterobacter asburiae* QF11 Isolated from *Chenopodium quinoa* Willd. Microorganisms.

[2] Lin S.M., Baek C.Y., Jung J.H. (2020). Antioxidant Activities of an Exopolysaccharide (DeinoPol) Produced by the Extreme Radiation-Resistant Bacterium *Deinococcus radiodurans*. Sci. Rep..

[3] Freitas F., Alves V.D., Reis M.A.M. (2011). Advances in bacterial exopolysaccharides: From production to biotechnological applications. Trends Biotechnol..

[4] Freitas F.D., Alves V., Pais J., Carvalheira M., Costa N., Oliveira R.M., Reis M.A.M. (2010). Production of a new exopolysaccharide (EPS) by *Pseudomonas oleovorans* NRRL B-14682 grown on glycerol. Process. Biochem..

[5] Angelin J., Kavitha M. (2020). Exopolysaccharides from probiotic bacteria and their health potential. Int. J. Biol. Macromol..

[6] Liang T.-W., Tseng S.-C., Wang S.-L. (2016). Production and Characterization of Antioxidant Properties of Exopolysaccharide(s) from *Peanibacillus mucilaginosus* TKU032. Mar. Drugs.

[7] Vu B., Chen M., Crawford R.J., Ivanova E.P. (2009). Bacterial Extracellular Polysaccharides Involved in Biofilm Formation. Molecules.

[8] Radchenkova N., Tomova A., Kambourova M. (2014). Biosynthesis of an Exopolysaccharide Produced by *Brevibacillus Thermoruber* 438. Biotechnol. Biotechnol. Equip..

[9] Kumar A.S., Mody K., Jha B. (2007). Bacterial exopolysaccharides—A perception. J. Basic Microbiol..

[10] Santos F.L., Amorim G.M. (2018). Biotechnological challenges and perspectives ofusing exopolysaccharides. J. Anal. Pharm. Res..

[11] Caggianiello G., Kleerebezem M., Spano G. (2016). Exopolysaccharides produced by lactic acid bacteria: From health-promoting benefits to stress tolerance mechanisms. Appl. Microbiol. Biotechnol..

[12] Yu Y.J., Chen Z., Chen P.T., Ng I.-S. (2018). Production, characterization and antibacterial activity of exopolysaccharide from a newly isolated *Weissella cibaria* under sucrose effect. J. Biosci. Bioeng..

[13] Shao L., Wu Z., Zhang H., Chen W., Ai L., Guo B. (2014). Partial characterization and immunostimulatory activity of exopolysaccharides from *Lactobacillus rhamnosus* KF5. Carbohydr. Polym..

[14] Ghosh T., Chattopadhyay K., Marschall M., Karmakar P., Mandal P., Ray B. (2009). Focus on antivirally active sulfated polysaccharides: From structure-activity analysis to clinical evaluation. Glycobiology.

[15] Takeuchi A., Kamiryou Y., Yamada H., Eto M., Shibata K., Haruna K., Naito S., Yoshikai Y. (2009). Oral administration of xanthan gum enhances antitumor activity through Toll-like receptor 4. Int. Immunopharmacol..

[16] Patel S., Majumder A., Goyal A. (2012). Potentials of exopolysaccharides from lactic acid bacteria. Indian J. Microbiol..

[17] Prete R., Alam M.K., Perpetuini G., Perla C., Pittia P., Corsetti A. (2021). Lactic Acid Bacteria Exopolysaccharides Producers: A Sustainable Tool for Functional Foods. Foods.

[18] Senoner T., Dichtl W. (2019). Oxidative Stress in Cardiovascular Diseases: Still a Therapeutic Target?. Nutrients.

[19] Yang S., Lian G. (2020). ROS and diseases: Role in metabolism and energy supply. Mol. Cell Biochem..

[20] Chiste R.C., Freitas M., Mercadante A.Z., Fernandes E. (2015). Superoxide Anion Radical: Generation and Detection in Cellular and Non-Cellular Systems. Curr. Med. Chem..

[21] Gulcin İ. (2020). Antioxidants and antioxidant methods: An updated overview. Arch. Toxicol..

[22] Ilyas N., Mumtaz K., Akhtar N., Yasmin H., Sayyed R.Z., Khan W., Enshasy H.A.E., Dailin D.J., Elsayed E.A., Ali Z. (2020). Exopolysaccharides Producing Bacteria for the Amelioration of Drought Stress in Wheat. Sustainability.

[23] Šovljanski O., Pezo L., Stanojev J., Bajac B., Kovač S., Tóth E., Ristić I., Tomić A., Ranitović A., Cvetković D. (2021). Comprehensive Profiling of Microbiologically Induced CaCO_3_ Precipitation by Ureolytic *Bacillus* Isolates from Alkaline Soils. Microorganisms.

[24] Wang S., Hou Q., Guo Q., Zhang J., Sun Y., Wei H., Shen L. (2020). Isolation and Characterization of a Deoxynivalenol-Degrading Bacterium *Bacillus licheniformis* YB9 with the Capability of Modulating Intestinal Microbial Flora of Mice. Toxins.

[25] Biswas J.K., Banerjee A., Sarkar B., Sarkar D., Sarkar S.K., Rai M., Vithanage M. (2020). Exploration of an Extracellular Polymeric Substance from Earthworm Gut Bacterium (*Bacillus licheniformis*) for Bioflocculation and Heavy Metal Removal *Potential*. Appl. Sci..

[26] Banoon S., Ali Z., Salih T. (2020). Antibiotic resistance profile of local thermophilic *Bacillus licheniformis* isolated from Maysan province soil. Comun. Sci..

[27] Hirad A., Bahkali A., Khiyami M., Ahmed M., Santhapa C., Elgorban A., Al-Sum B. (2014). Bioactivity of Marine *Bacillus licheniformis* Ksawd3 Isolated from Arabian Gulf, Saudi Arabia. J. Pure Appl. Microbiol..

[28] Petrova P., Petlichka S., Petrov K. (2020). New *Bacillus* spp. with potential for 2,3-butanediol production from biomass. J. Biosci. Bioeng..

[29] Song C.W., Rathnasingh C., Park J.M., Lee J., Song H. (2018). Isolation and evaluation of *Bacillus* strains for industrial production of 2,3-butanediol. J. Microbiol. Biotechnol..

[30] Jurchescu I.M., Hamann J., Zhou X., Ortmann T., Kuenz A., Prusse U., Lang S. (2013). Enhanced 2,3-butanediol production in fed-batch cultures of free and immobilized *Bacillus licheniformis* DSM 8785. Appl. Microbiol. Biotechnol..

[31] Cheng Y.-H., Horng Y.-B., Chen W.-J., Hua K.-F., Dybus A., Yu Y.-H. (2021). Effect of Fermented Products Produced by *Bacillus licheniformis* on the Growth Performance and Cecal Microbial Community of Broilers under Coccidial Challenge. Animals.

[32] Hu Q., Fang Y., Zhu J., Xu W., Zhu K. (2021). Characterization of *Bacillus* Species from Market Foods in Beijing, China. Processes.

[33] Vinothkanna A., Sathiyanarayanan G., Balaji P., Mathivanan K., Pugazhendhi A., Ma Y., Sekar S., Thirumurugan R. (2021). Structural characterization, functional and biological activities of an exopolysaccharide produced by probiotic *Bacillus licheniformis* AG-06 from Indian polyherbal fermented traditional medicine. Int. J. Biol. Macromol..

[34] Xu Z., Chen G., Xue L., Zhang H., Wang J., Xiang H., Lia J., Zheng K. (2019). Isolation, structural characterizations and bioactivities of exopolysaccharides produced by *Bacillus licheniformis*. Int. J. Biol. Macromol..

[35] Kwon J.-H., Won S.-J., Moon J.-H., Lee U., Park Y.-S., Maung C.E.H., Ajuna H.B., Ahn Y.S. (2021). *Bacillus licheniformis* PR2 Controls Fungal Diseases and Increases Production of Jujube Fruit under Field Conditions. Horticulturae.

[36] Matei M.-C., Andrei S.M., Buza V., Cernea M.S., Dumitras D.A., Neagu D., Rafa H., Popovici C.P., Szakacs A.R., Catinean A. (2021). Natural Endotoxemia in Dogs—A Hidden Condition That Can Be Treated with a Potential Probiotic Containing *Bacillus subtilis*, *Bacillus licheniformis* and *Pediococcus acidilactici*: A Study Model. Animals.

[37] Zeng X., Li Q., Yang C., Yu Y., Fu Z., Wang H., Fan X., Yue M., Xu Y. (2021). Effects of *Clostridium butyricum*- and *Bacillus* spp.-Based Potential Probiotics on the Growth Performance, Intestinal Morphology, Immune Responses, and Caecal Microbiota in Broilers. Antibiotics.

[38] Arsov A., Petrov K., Petrova P. (2021). Enhanced activity by genetic complementarity: Heterologous secretion of clostridial cellulases by *Bacillus licheniformis* and *Bacillus velezensis*. Molecules.

[39] Tsigoriyna L., Ganchev D., Petrova P., Petrov K. (2021). Highly Efficient 2,3-Butanediol Production by *Bacillus licheniformis* via Complex Optimization of Nutritional and Technological Parameters. Fermentation.

[40] Petrov K., Petrova P. (2009). Isolation and Molecular Identification of *Klebsiella Pneumoniae* Strains Producing Diols from Glycerol. Biotechnol. Biotechnol. Eq..

[41] Eneva R., Engibarov S., Strateva T., Abrashev R., Abrashev I. (2011). Biochemical studies on the production of neuraminidase by environmental isolates of *Vibrio cholerae* non-O1 from Bulgaria. Canadian J. Microbiol..

[42] Okonkwo C.C., Ujor V., Ezeji T.C. (2017). Investigation of relationship between 2,3-butanediol toxicity and production during growth of *Paenibacillus polymyxa*. New Biotechnol..

[43] Petrova P., Petrov K. (2011). Antimicrobial activity of starch-degrading *Lactobacillus* strains isolated from Boza. Biotechnol. Biotechnol. Equip..

[44] Ilyasov I.R., Beloborodov V.L., Selivanova I.A., Terekhov R.P. (2020). ABTS/PP Decolorization Assay of Antioxidant Capacity Reaction Pathways. Int. J. Mol. Sci..

[45] Munteanu I.G., Apetrei C. (2021). Analytical Methods Used in Determining Antioxidant Activity: A Review. Int. J. Mol. Sci..

[46] Dubois M., Gilles K.A., Hamilton J.K., Rebers P.A., Smith F. (1956). Colorimetric method for determination of sugars and related substances. Anal. Chem..

[47] Nielsen S.S. (2017). Food Analysis Laboratory Manual, Food Science Text Series.

[48] Bradford M.M. (1976). A rapid and sensitive method for the quantitation of microgram quantities of protein utilizing the principle of protein-dye binding. Anal. Biochem..

[49] Hu X., Pang X., Wang P.G., Chen M. (2018). Isolation and Characterization of an Antioxidant Exopolysaccharide Produced by *Bacillus* sp. S-1 from Sichuan Pickles. Carbohydrate Polym..

[50] Jia K., Tao X., Liu Z., Zhan H., He W., Zhang Z., Zeng Z., Wei H. (2019). Characterization of novel exopolysaccharide of *Enterococcus faecium* WEFA23 from infant and demonstration of its in vitro biological properties. Int. J. Biol. Macromol..

[51] Parveen R., Anandharaj M., David A. (2018). Characterization of a novel exopolysaccharide produced by *Lactobacillus gasseri* FR4 and demonstration of its in vitro biological properties. Int. J. Biol. Macromol..

[52] Min W.-H., Fang X.-B., Wu T., Fang L., Liu C.-L., Wang J. (2019). Characterization and antioxidant activity of an acidic exopolysaccharide from *Lactobacillus plantarum* JLAU103. J. Biosci. Bioeng..

[53] Zhu Y., Wang C., Jia S., Wang B., Zhou K., Chen S., Yang Y., Liu S. (2018). Purification, Characterization and Antioxidant Activity of the Exopolysaccharide From *Weissella Cibaria* SJ14 Isolated From Sichuan Paocai. Int. J. Biol. Macromol..

[54] Asgher M., Rani A., Khalid N., Qamar S.A., Bilal M. (2021). Bioconversion of sugarcane molasses waste to high-value exopolysaccharides by engineered *Bacillus licheniformis*. Case Studies Chem. Environ. Eng..

[55] Dogan N.M., Doganli G.A., Dogan G., Bozkaya O. (2015). Characterization of Extracellular Polysaccharides (EPS) Produced by Thermal *Bacillus* and Determination of Environmental Conditions Affecting Exopolysaccharide Production. Int. J. Environ. Res..

[56] Van Dyk J.S., Low Ah Kee N., Frost C.L., Pletschke B.I. (2012). Extracellular polysaccharide production in *Bacillus licheniformis* SVD1 and its immunomodulatory effect. BioResources.

[57] Asgher M., Uroojm Y., Qamar S.A., Khalid N. (2020). Improved exopolysaccharide production from *Bacillus licheniformis* MS3: Optimization and structural/functional characterization. Int. J. Biol. Macromol..

[58] Fan Y., Wang J., Gao C., Zhang Y., Du W. (2020). A novel exopolysaccharide-producing and long-chain n-alkane degrading bacterium *Bacillus licheniformis* strain DM-1 with potential application for *in-situ* enhanced oil recovery. Sci. Rep..

[59] Spanò A., Gugliandolo C., Lentini V., Maugeri T.L., Anzelmo G., Poli A., Nicolaus B. (2013). A Novel EPS-Producing Strain of *Bacillus licheniformis* Isolated from a Shallow Vent off Panarea Island (Italy). Curr. Microbiol..

[60] Abdalla A.K., Ayyash M.M., Olaimat A.N., Osaili T.M., Al-Nabulsi A.A., Shah N.P., Holley R. (2021). Exopolysaccharides as Antimicrobial Agents: Mechanism and Spectrum of Activity. Front. Microbiol..

[61] Riaz Rajoka M.S., Jin M., Haobin Z., Li Q., Shao D., Jiang C., Huang Q., Yang H., Shi J., Hussain N. (2018). Functional characterization and biotechnological potential of exopolysaccharide produced by *Lactobacillus rhamnosus* strains isolated from human breast milk. LWT Food Sci. Technol..

[62] Abinaya M., Vaseeharan B., Divya M., Vijayakumar S., Govindarajan M., Alharbi N.S., Khaled J.M., Al-anbr M.N., Benelli G. (2018). Structural characterization of *Bacillus licheniformis* Dahb1 exopolysaccharide—Antimicrobial potential and larvicidal activity on malaria and Zika virus mosquito vectors. Environ. Sci. Pollut. Res..

[63] Cholera. https://www.who.int/news-room/fact-sheets/detail/cholera.

[64] Li S., Huang R., Shah N.P., Tao X., Xiong Y., Wei H. (2014). Antioxidant and antibacterial activities of exopolysaccharides from *Bifidobacterium bifidum* WBIN03 and *Lactobacillus plantarum* R315. J. Dairy Sci..

[65] Song Y.R., Song N.E., Kim J.H., Nho Y.C., Baik S.K. (2011). Exopolysaccharide produced by *Bacillus licheniformis* strain isolated from Kimchi. J. Gen. Appl. Microbiol..

[66] Almeida M.M.B., Sousa P.H.M., Arriaga A.M.C., Prado G.M., Magalhães C.E.C., Maia G.A., Lemos T.L.G. (2011). Bioactive compounds and antioxidant activity of fresh exotic fruits from northeastern Brazil. Food Res. Int..

